# Evaluation of Microwave Steam Bags for the Decontamination of Filtering Facepiece Respirators

**DOI:** 10.1371/journal.pone.0018585

**Published:** 2011-04-15

**Authors:** Edward M. Fisher, Jessica L. Williams, Ronald E. Shaffer

**Affiliations:** National Personal Protective Technology Laboratory, National Institute for Occupational Safety and Health, Pittsburgh, Pennsylvania, United States of America; Cochrane Acute Respiratory Infections Group, Italy

## Abstract

Reusing filtering facepiece respirators (FFRs) has been suggested as a strategy to conserve available supplies for home and healthcare environments during an influenza pandemic. For reuse to be possible, used FFRs must be decontaminated before redonning to reduce the risk of virus transmission; however, there are no approved methods for FFR decontamination. An effective method must reduce the microbial threat, maintain the function of the FFR, and present no residual chemical hazard. The method should be readily available, inexpensive and easily implemented by healthcare workers and the general public. Many of the general decontamination protocols used in healthcare and home settings are unable to address all of the desired qualities of an efficient FFR decontamination protocol. The goal of this study is to evaluate the use of two commercially available steam bags, marketed to the public for disinfecting infant feeding equipment, for FFR decontamination. The FFRs were decontaminated with microwave generated steam following the manufacturers' instructions then evaluated for water absorption and filtration efficiency for up to three steam exposures. Water absorption of the FFR was found to be model specific as FFRs constructed with hydrophilic materials absorbed more water. The steam had little effect on FFR performance as filtration efficiency of the treated FFRs remained above 95%. The decontamination efficacy of the steam bag was assessed using bacteriophage MS2 as a surrogate for a pathogenic virus. The tested steam bags were found to be 99.9% effective for inactivating MS2 on FFRs; however, more research is required to determine the effectiveness against respiratory pathogens.

## Introduction

The potential reuse of National Institute for Occupational Safety and Health (NIOSH) -certified N95 filtering facepiece respirators (FFRs) has been suggested as a possible strategy to conserve available supplies for home and healthcare environments during an influenza pandemic [1], [2]. Reuse of FFRs may result in a risk of contact transmission by touching a contaminated surface of the respirator followed by touching the eyes, nose, and/or mouth. Physical and chemical methods to remove or inactivate viruses on FFR surfaces have been previously examined [3], [4], [5], [6], [7], [8], [9], [10], [11]. These methods were evaluated for decontamination efficacy, effect on FFR filtration and fit, wearer safety (i.e. chemical residues and off-gassing) and processing cost as suggested in a report issued by the Institute of Medicine (IOM) [1]. The IOM report also recommended that simple decontamination methods should be evaluated for ease of implementation in home and healthcare settings.

Some of the previously examined methods, although promising in laboratory studies, may not be universally suited for both healthcare and home environments. Methods that require decontamination equipment such as UV lights, vaporous hydrogen peroxide generators, and moist heat incubators would be better suited for healthcare facilities where such disinfection equipment is more likely to be available. Home environments lack sophisticated decontamination technology, but have disinfectants such as bleach and peroxide; however, the use of these products for FFR decontamination would require customized procedures which may not be easily executed by the general public. Moreover, skin and inhalation health hazards from the use of chemically treated FFRs are a concern [8], [11]. Healthcare professionals, including infection control practitioners, are better prepared to follow customized detailed disinfection procedures than the general public due to training and experience. The logistics of an FFR decontamination program also need to be considered. FFR decontamination in healthcare settings may occur as a batch process, whereby one or a few employees decontaminate all FFRs or as an individual process, whereby the individual user is responsible for decontaminating their own respirator. Each scenario requires a system to identify the FFR user (to avoid sharing of FFRs among users), to track the number of decontamination cycles for each FFR, and to provide a means to efficiently store the FFR between uses.

One possibility of overcoming the problems posed by the lack of decontamination equipment and elaborate protocols is to use technology that is readily available and already used by the general public for other applications with similar requirements. Off-the-shelf microwave steam bags (MSBs) are one option that may be used in healthcare and home environments. These bags, typically used to decontaminate breast pump and infant feeding accessories, are available for purchase in many retail stores where infant associated goods are sold. The instructions are written for the general public and are based on the operation of a microwave oven, which are readily available in home and healthcare environments. The use of commercially available steam bags for FFR decontamination has not been investigated, although previous studies suggest that microwave generated steam decontamination is promising [3], [5], [6], [12], [13]. The goal of this study is to evaluate the use of two commercially available steam bags for FFR decontamination with specific considerations to FFR filtration performance, FFR water absorption, decontamination efficacy, ease of use, and logistic benefits.

## Results

Table 1 lists the water absorption/retention and filtration efficiency of all FFR models after one cycle of steam bag decontamination using the MSB X bags. All of the six FFR models (one sample per model) surpassed the filtration efficiency requirements of 95%. The absorption values for models 3M 1860, 3M 8210 and the Cardinal Health N95 were roughly an order of magnitude higher than the values for 3M 1870, Kimberly-Clark PFR95, and Moldex 2200. The models 3M 1860, 3M 8210 and the Cardinal Health N95 remained wet after the 60 min drying period and were eliminated from further testing.

**Table 1:** Phase 1 screening of FFRs for water absorbency and filtration efficiency.

FFR Details			Water Content (g) #		Filtration Efficiency (%)	
Model	Type	Contains Hydrophilic layer(s)*	After decon.	60 min	As received	After 1X
3M 1870	Surgical	no	0.4	0.1	99.67	99.62
3M 1860	Surgical	yes	13.5	9.6	99.28	99.47
KC PFR95	Surgical	no	0.9	0	96.13	95.77
3M 8210	Particulate	yes	11.6	8.2	99.88	99.34
Cardinal Health	Particulate	yes	12.8	11.2	99.62	99.56
Moldex 2200	Particulate	no	1.5	0.2	98.52	99.24

*Data modified from references (10) and (14).

# Determined using MSB X bags.

n = 1.

In the second phase of testing, the triplicate samples for each of the FFR models, 3M 1870, Kimberly-Clark PFR95, and Moldex 2200, passed the filtration efficiency testing after three cycles of decontamination using both steam bag brands (Table 2). For the MSB X bags, the filtration efficiencies of the experimental models were statistically similar to the controls for both the 3M 1870 (p = 0.19) and the Moldex 2200 (p = 0.40), while the treated Kimberly-Clark PFR95 models were statistically different from the controls (p = 0.01). MSB Y bags produced statistically similar results for the control and treated samples for each model; 3M 1870 (p = 0.19) Moldex 2200 (p = 0.40) and Kimberly-Clark PFR95 (p = 0.42). The results for drying of the FFRs were similar for 30 min compared to the 60 min drying time (Tables 1 and 2). All models from the second phase of testing were included in the third phase of testing.

**Table 2:** Phase 2 testing for water absorbency and filtration efficiency.

FFR		Water Content (g) #		Filtration Efficiency (%)		
Type	Model	After (3X) decon.	30 min	As received	MSB X (3X)	MSB Y (3X)
Surgical	3M 1870	1.7±1.4	0.1±0.1	99.7±0.1	98.6±0.6	99.0±1.1
Surgical	KC PFR95	1.3±1.3	0±0	96.1±0.4	95.5±0.3	96.4±1.2
Particulate	Moldex 2200	0.9±0.4	0.1±0.1	98.5±1.0	98.6±0.8	98.4±1.5

# Determined using MSB X bags.

n = 3.

Table 3 lists the CV values for the MS2 contamination of each FFR model. Five of the six data sets achieved the ASTM E2721-10 quality objective CV value of ≤40% [18]. The average decontamination efficacy resulting from the use of MSB X bags was greater than 99.9% (3 logs) for all three FFR models tested (Table 3). The average decontamination efficacy for the Moldex model was greater than 99.99% or 4 logs. The MS2 challenge concentration for the Moldex models was more than 2 logs higher than the Kimberly Clark (7.1) or 3M 1860 (7.6). MSB Y bags achieved 99.9% reduction of MS2 for two FFR models while the results of the third model measured greater than or equal to 99.86%.

**Table 3:** Decontamination efficacy of the microwave steam bags.

	FFR Model	MS2 from load controls *	CV (%)	MS2 from Steam Treated FFR *	Difference (Load vs. Treated) *	Reduction (%)
**MSB X**	**1870**	7.57±0.08	18.5	4.47±0.32	3.10	99.90
	**KC**	7.09±0.17	37.6	3.85±0.35	3.25	99.93
	**Moldex**	9.96±0.06	14.6	5.32±0.30	4.64	99.99
**MSB Y**	**1870**	6.93±0.16	32.8	≤3.69#	≥3.24	≥99.94
	**KC**	8.15±0.25	62.3	4.70±0.69	3.45	99.93
	**Moldex**	7.04±0.09	19.8	≤3.93#	≥3.11	≥99.86

* Values in Log_10_ (pfu/FFR).

# Two of three trials reached detection limits.

## Discussion

**Figure 1:**
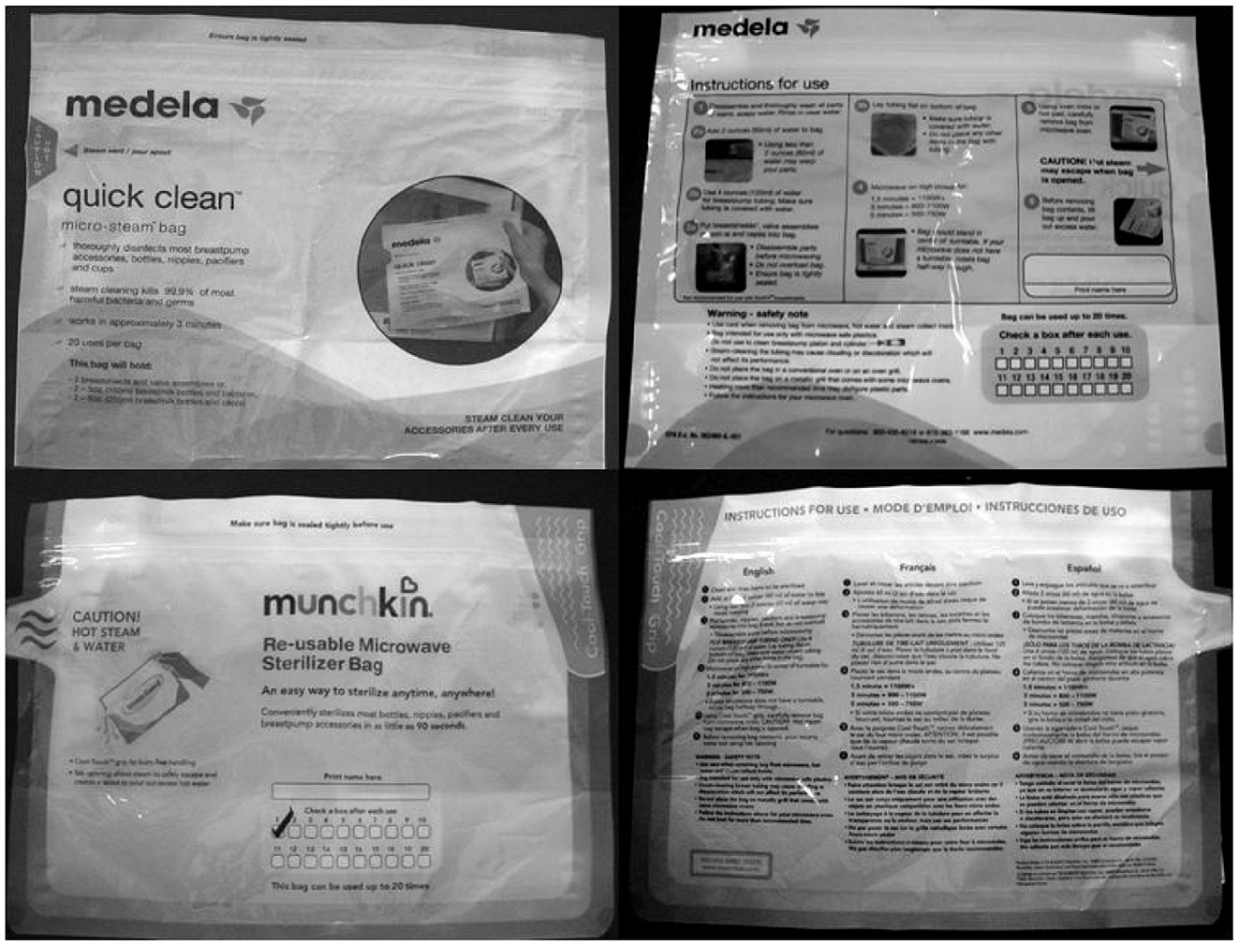
Photographs of the front (left) and back (right) panels of the microwave steam bags. Top: MSB X bags. Bottom: MSB Y bags.

Commercially available MSBs offer intrinsic benefits for FFR decontamination in home and healthcare settings. The steam bags, constructed for the purpose of disinfection (baby bottles and breast pumps), are readily available for purchase. The instructions for use are clearly provided on the side of the bags (Fig. 1). Simple, well-illustrated decontamination instructions are important for users with limited experience in disinfection and sterilization. For the MSB X bags, the instructions are included in an approximate 8” ×4” panel and are accompanied by step-by-step photographs. MSB Y bags include use instruction in three languages, English, Spanish, and French. The instructions are written for a range of microwave powers (500–1100W+), providing versatility for multiple microwave models. The steam bag can provide a dual function of storage and decontamination. A used FFR can be stored in the bag and decontaminated when use is required. Defined areas on the bag for the user's name and a checkbox indicating the number of uses provides a method for inventory accounting.

Steam sterilization, by the use of autoclaves, is routinely used in the processing of medical equipment. Autoclaves produce high pressure saturated steam and are effective at inactivating microorganisms including spores [20]. Unfortunately, autoclaving is highly destructive process for some FFR models [9]. Atmospheric applications of steam, such as the use of MSBs, are less destructive on FFRs but are less effective in inactivating microorganisms. Furthermore, the disinfecting ability of steam bags is not well characterized. Labeling on the steam bags or the steam bag packaging claims that “steam kills 99.9% of most harmful bacteria and germs”. The steam bags repeatedly produced a 3 log or 99.9% reduction in MS2 for the three FFR models tested in this study (Table 3). FFR decontamination using microwave generated steam has been examined previously, although without the use of a steam bag. Fisher et al., 2009, demonstrated a greater than 4 log, or 99.99%, reduction of MS2 virus after a 45 s treatment [3]. This study was performed on small FFR coupons contaminated with MS2-containing droplet nuclei in the same model microwave used in this current investigation. Fisher et al. (2010) examined cyclic MS2 contamination and decontamination of FFRs with microwave generated steam [5]. The findings in that study suggest that protective residues (proteins, respiratory secretions, cellular debris, etc.), which are a component of infectious aerosols, have less of an effect on the decontamination efficacy of steam compared to other decontamination methods. Heimbuch et al. studied the use of microwave generated steam on the inactivation of H1N1 deposited on FFRs as aerosols and droplets [6]. The microwave generated steam yielded a >4-log reduction of viable H1N1 virus for all FFR tested. In 93% of the experiments, the virus was reduced to levels below the limit of detection of the method. The presence of some viable virus on the FFRs was speculated to be “due to non-uniform distribution of steam over the entire surface of the FFR”. Heimbuch et al. further speculated that “optimization of the water reservoir holder will likely minimize or eliminate this issue”. The steam bags, used in this study, provide a defined volume for the entrapment of steam and, therefore, a more uniform application.

The FFR filtration performance for the three cycle treatments was within acceptable levels of the selection criterion for each FFR model treated in each steam bag brand. Bergman et al. reported similar results with no deleterious effect of microwave generated steam on the filtration performance of three surgical and three particulate N95 FFRs [12]. Moreover Bergman et al. and Viscusi et al. found fit of the FFR models used in their investigations to be unaffected by the use of microwave generated steam [12], [21]. Although the steam bags used in this study differs from the vessel used to house the FFRs in the Viscusi and Bergman studies, the results are promising.

The use of a steam bag has some limitations for FFR decontamination. The steam bags do not compartmentalize the water reservoir and sample location. The FFR is placed directly into the water in the reservoir, which produces the potential for water absorption by the FFR material. Water absorbency is important as a saturated FFR would require an extended drying period before reuse is possible. An extended drying period is counterproductive to increasing FFR supply in the event of shortages due to high demand. The potential to use the steam bags for FFR decontamination will likely be FFR model specific, as demonstrated by the water absorption data in this study (Tables 1 and 2). The absorption characteristics were also independent of the FFR classification as a particulate or surgical mask, which suggests simplification of determining decontamination potential is unlikely. These findings are supported by the results discussed in Viscusi et al. 2009, where differences in the hydrophobicity of FFR models, individual layers of FFRs, and even differences between the surfaces of a given layer were confirmed [8]. In fact, the FFR models eliminated after the first phase of testing were found to contain at least one hydrophilic layer, whereas the models proceeding to the second phase of testing were constructed entirely of hydrophobic materials [8], [22].

The decontamination procedure and demonstrated efficacy of the MSBs are not in alignment with current FDA guidelines and requirements for the reuse of single use medical devices [20], [23], [24]. However, government recommendations for FFR reuse are complicated; CDC and NIOSH recommendations have permitted reuse (i.e., multiple donnings of a previous worn FFR) without decontamination in some unique situations such treating TB patients and in emergency situations when supplies are limited (e.g., during the 2009–10 novel H1N1 influenza pandemic) [25], [26]. Furthermore, many FFR models used in healthcare (including 3 in this study) and nearly half of those models in the U.S. Strategic National Stockpile are not currently regulated by FDA as they are not being marketed as medical devices. Currently, NIOSH respirator certification does not include provisions for FFR decontamination and reuse. Decontamination of NIOSH certified FFRs for purposes of reuse is not recommended in the workplace, primarily because of concerns that decontamination would degrade the performance of the respirator. Thus, the results of this study should be viewed from the context of informing future government, public health, and infection control recommendations in an emergency, rather than as recommending changes to routine practice.

A cleaning procedure was not included as part of this study; however, it was previously demonstrated that soil load accumulation may not significantly impact microwave generated steam decontamination of FFRs [5]. Likewise, a high level disinfection, which eliminates all microorganisms except for a small number of bacterial spores, was not achieved with the use of the steam bags. Initial virus titers between 10^7^ and 10^10^ pfu/FFR were reduced by 99.9%; leaving roughly 10^4^ to 10^6^ pfu/FFR. The titer of viable MS2 remaining on the respirator can present major health hazard concerns. However, the number of viable MS2 applied to the respirators (7–10 log_10_ pfu) greatly exceeds the expected contamination levels of in-use scenarios.

In healthcare and home environments alike, the performance of the steam, and microwave ovens, may demonstrate some variability. It should be noted that the microwave used in this study was rated at 1100 W by the manufacturer, but was experimentally determined to function at 750 W previously [9]. It is possible that applying a longer treatment time, indicated by the steam bag instructions for a 750 W microwave, would produce increased decontamination efficacy. However, this introduces another level of complexity as it is possible that microwave performance in homes and healthcare settings may not be consistent with manufacturer ratings. It is possible that some of the steam bags may demonstrate inconsistent behavior. In this limited investigation, the steam bags were monitored for failures in the seams and zip lock seals after decontaminations with no discernable failures. The consistent decontamination performance of the steam bags supports the observation of maintained structural integrity during the steam procedure. However, care must be taken not to generalize this finding beyond the scope of this study.

More studies are required before the use of steam bags can be considered for FFR decontamination for the purpose of reuse. In general, reuse requires a higher degree of rigor than single use applications. Commonly used in the evaluation of medical devices, an ultrastructural analysis of the decontaminated FFRs may help to address concerns and knowledge gaps associated with FFR reuse. Quality control assessments of the steam bags and microwave ovens should be performed to investigate the utility of the steam bag decontamination procedure. Likewise, implementing MSB decontamination of FFRs in home and healthcare settings would present quality control issues which should be investigated for each environment. The use of MS2, a nonenveloped virus, in this study does not accurately reflect the potential efficacy of the steam bag against enveloped viruses including 2009 H1N1. In general, enveloped viruses are more susceptible to decontamination due to the fragile lipid coat. Testing the steam bags against other microbes can assist discerning the steam bags' potential. FFRs decontaminated using the steam bags should be fit tested to ascertain if any changes to FFR shape and fit occurred as a result of the steam process, although previous research with microwave generated steam suggests that this is unlikely.

## Materials and Methods

### Experimental procedure

The evaluation of the feasibility of steam bag decontamination of FFRs was studied in three phases. In the first phase, a preliminary screening of the six models of respirators treated in one brand of MSB was conducted using two quality objectives: limited filtration performance degradation and low water absorbency/retention. Each quality objective was evaluated using a predetermined standard. Firstly, the steam bag decontamination must not degrade the filtration performance of the FFR below the efficiency required (95% efficient) by NIOSH certification requirements outlined in 42 CFR 84. Secondly, the FFR must be dry (defined for this study as less than 1 g water content) within 60 min of drying time under room conditions (approx. 20°C and 60% RH). The rationale for this requirement is the users would be unlikely to find wearing a wet respirator to be uncomfortable, previously identified as a barrier to respirator tolerability [14], [15]. The filtration efficiency and water absorbency/retention determination was performed for one sample of each FFR model for phase 1.

In phase 2 testing, FFR models passing the preliminary assessment were evaluated for filtration efficiency following three cycles of steam bag sterilization which included a 30 min drying period between treatments. Each model was evaluated in triplicate for each MSB brand. The FFRs were evaluated for water absorption/retention after 30 min of drying time following steam treatment using one MSB brand. FFR models exceeding the predefined quality standards were eliminated from phase 3 testing.

In the final phase of testing, the decontamination efficacy of the steam bag was determined in triplicate for the FFR models passing the phase 2 evaluation using both brands of bags. For each FFR model, six samples were contaminated with MS2 droplets. The MS2 from three of the samples for each FFR model was collected and enumerated to determine the loading level. The other three samples were decontaminated using one brand of MSBs. The process for each FFR was repeated for the second brand of MSBs. Upon decontamination the MS2 was collected from the filter samples and enumerated via plaque assay.

### Respirator selection

Six respirator models were used in this study. Three of the models, namely the 3M 1870 (3M, St. Paul MN), 3M 1860 (3M, St. Paul MN), and the Kimberly-Clark PFR95 (Kimberly-Clark, Dallas, TX) are surgical N95 FFRs. Surgical N95 FFRs are NIOSH-approved particulate respirators that have also been cleared by the Food and Drug Administration (FDA) as medical devices. Three particulate FFR models included in the study are the 3M 8210 (3M, St. Paul MN), Moldex 2200 (Moldex, Culver City, CA) and Cardinal Health (Cardinal Health, Dublin, OH). All models used were available in the Strategic National Stockpile at the time of writing except for the Cardinal Health FFR, which was randomly selected from the laboratory stock.

### Steam bag design and use instructions

**Figure 2:**
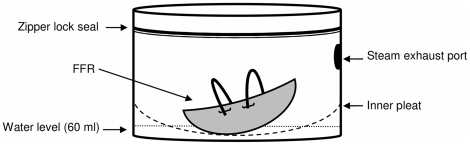
Illustration of the components of the microwave steam bags and the placement of a typical FFR into the water reservoir.

Figure 1 shows the front and back panels of the two brands of MSBs used for this study, namely, the Medela Quick Clean^TM^ MICRO-STEAM^TM^ BAGS (Medela, McHenry, IL) and the Munchkin_®_ Steam Guard™ Bags (Munchkin Inc., North Hills, CA). These bags will be denoted as “MSB X” or “MSB Y” for the former and later, respectively. Both steam bag brands have similar design structures which include a zipper lock seal, a steam exhaust port, internal pleat, and a volume of approximately 2.2 L (Fig. 2).

The manufacturer's instructions for use with baby feeding accessories were applied to the steam treatment of FFRs. The instructions were the same for each steam bag brand. Individual FFRs were placed inside separate bags filled with 60 ml of tap water (Fig. 2). The bags were sealed, using the bag's integrated zipper lock seal and placed in a commercially available Sharp Model R-305KS (2450 MHz, 1100 W) microwave oven (Sharp Electronics, Mahwah, NJ, USA). The FFRs in the sealed steam bags were irradiated on high power for 90 s; the prescribed time for a microwave with a rating of 1100 W.

### Filtration performance

A Model 8130 Automated Filter Tester (AFT) (TSI, Inc., St. Paul, MN, USA) was used to measure initial percent filter aerosol penetration and filter airflow resistance for FFR models as received (control), 1 cycle treated FFRs, and 3 cycle treated FFRs. The TSI 8130 AFT delivers a solid polydispersed sodium chloride (NaCl) aerosol that meets the particle size distribution criteria set forth in 42 CFR 84 Subpart K, Section 84.181 for NIOSH certification (CFR, 1995). Filter penetration testing was performed using a similar but abbreviated version of the NIOSH certification protocol previously used to evaluate FFR filtration performance [8], [9], [16].

### Water absorbency determination

FFRs were decontaminated using the MSB X bags as described above. The FFRs were weighed prior to decontamination to determine the dry weight and reweighed immediately following decontamination and after a predetermined drying period of 30 or 60 min to determine the wet weight. The dry weight was subtracted from the wet weight to determine the amount of water absorbed or retained by the material of the FFR. MSB Y bags were not used to assess FFR water absorbency; however, comparable water absorbency values for FFRs treated in the in both brands of bags are expected due to the similar steam bag designs, which have the FFR partially submerged in the water (Fig. 2).

### Media, virus, and host cells

The media, virus, and host cells used in this research have been described previously [3]. Briefly, American Type Culture Collection (ATCC) medium 271 (http://www.atcc.org/Attachments/3600.pdf) was used to grow *Escherichia coli* (ATCC 15597) and prepare, store, recover, aerosolize and assay of MS2 (ATCC 1597-B1). The droplet-generating medium consisted of 100% ATCC medium 271. ATCC medium 271 amended with 5 g/L agar (Sigma-Aldrich, St. Louis, MO) was used to enumerate MS2 using a single agar plaque assays similar to methods previously described [3], [17].

### FFR virus droplet loading

**Figure 3:**
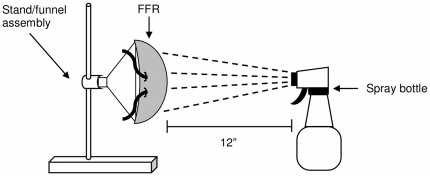
Schematic of the droplet loading method.

Virus containing droplets were applied to FFRs using a spray bottle (Fisherbrand Adjustable-Spray Mini-Wash Bottle, Fisher Scientific, Pittsburgh, PA). FFRs were mounted (friction fitted) to a funnel, which served as the FFR holder, and attached to a ring stand (Fig. 3). Models that were unable to fit the funnel were placed on a head form. The spray bottle, containing 100 ml of MS2 suspension (10^9^ plaque forming units/ml), was placed 12” from the closest plane of the FFR. Five sprays of virus containing droplets were applied to FFR. The contaminated FFRs were allowed to dry for 30 min. before decontamination. The funnel served as the preferred respirator holder since the entire FFR/funnel assembly could be placed in a rack to dry without handling the FFR.

### Data analysis and statistics

Filtration efficiencies for the FFR models were calculated from the percent aerosol penetration values (*%P*) generated by the TSI Model 8130 AFT (*filtration efficiency  = 100 - %P*). Control and experimental FFR filtration efficiencies were compared using a *t*-Test: Paired Two Sample for Means (Microsoft Excel 2007).

The repeatability of the MS2 application technique (spray bottle) used to contaminate the FFRs was assessed using ASTM Standard E2721-10 [18]. For each FFR model within each steam bag group (MSB X and MSB Y), the coefficient of variation (CV) of MS2 contamination was calculated as the ratio of the standard deviation to the mean plaque forming units (pfu) per respirator and expressed as a percentage. A CV ≤40% is the quality objective for contaminating materials described in ASTM Standard E2721. The same quality objective was used to evaluate a sophisticated device to apply virus droplets to FFRs [19]. That study found that CV values of <40% were achievable, but FFR design characteristics (shape, size, flexibility) affect repeatability.

The antiviral activity of the steam bags was determined for each FFR model by comparing the average log_10_ pfu of MS2 loaded onto three untreated (control) FFR samples with three steam-treated FFR samples. Percent reduction was also calculated for a given FFR model by dividing the number of recovered pfu from a treated respirator by the average pfu recovered from the untreated controls. The quotients calculated using the three treated samples were averaged to give average percent reduction.
